# Antibiotic prescribing practice in management of cough and/or diarrhoea in Moshi Municipality, Northern Tanzania: cross-sectional descriptive study

**Published:** 2012-08-13

**Authors:** Judith John Gwimile, Seif Abdallah Shekalaghe, Gibson Nsokolo Kapanda, Elton Richard Kisanga

**Affiliations:** 1 Department of Paediatric and Child Health, Kilimanjaro Christian Medical University College, P. O. Box 2240, Moshi, Tanzania; 2 Ifakara Health Institute, P.O.Box 74, Bagamoyo, Coastal Region, Tanzania; 3 Department of Community Medicine,Kilimanjaro Christian Medical University College, P. O. Box 2240, Moshi, Tanzania; 4 Department of Pharmacology, Kilimanjaro Christian Medical University College, Kilimanjaro Clinical Research Institute (KCRI), P. O. Box 3010, Moshi, Tanzania

**Keywords:** Antibiotics use, irrational prescribing, antibiotic prescribing, pneumonia, cough, diarrhea, under-five

## Abstract

**Introduction:**

The increase in resistance of many pathogens to currently available antibiotics has been recognized as life-threatening problem. The development of drug resistance is promoted by irrational prescribing behavior. Inappropriate use of antibiotics is attributed by over-prescription, inadequate dosage and use for non-bacterial infections. The purpose of this study was to assess antibiotic prescribing practices in the management of diarrhoea and cough among children attending hospitals in Moshi municipal, Tanzania.

**Methods:**

We conducted a cross-sectional descriptive hospital based study, from September 2010 to March 2011. All children presenting with diarrhoea and cough, aged between one month and 5years attended at the two hospitals were enrolled. Data were collected by a standard questionnaire. Information on the prescribed drugs was obtained from patient files.

**Results:**

A total of 384 children were enrolled. Of these, 326 (84.9%) received antibiotics; common prescribed antibiotics were penicillins, sulphonamides, aminoglycosides and macrolides. Eighty percent of children with acute watery diarrhoea and 68.9% with common cold were given antibiotics inappropriately. Inappropriate antibiotic prescription was significantly associated with prescriber being a clinical officer and assistant medical officer, and child having diarrhoea. Inappropriate antibiotic dosage was significantly occurred when prescriber was clinical officer with reference to medical officer.

**Conclusion:**

This study observed a high antibiotic prescription rate by clinicians and treatment guidelines for management of patients who presented with cough and/or diarrhoea are followed. Continuing professional development programmes for clinicians on prescription would help in reducing irrational prescribing practices.

## Introduction

Irrational use of antibiotics has been documented all over the world. World Health Organization (WHO) estimates that more than half of all medicines are prescribed, dispensed or sold inappropriately, and that half of all patients fail to take them correctly. Incorrect use may take the form of overuse, underuse and misuse of prescription or non-prescription medicines [1]. Rational use of medicines means that “patients receive medication appropriate to their clinical needs, in doses that meet their own individual requirements, for an adequate period of time, and at the lowest cost to them and their community” [2, 3]. Irrational use includes use of antibiotics for non-bacterial illnesses and non adherence to recommended dosing regimens, hence preventing desired therapeutic outcomes from being achieved and potentially increasing antimicrobial resistance [1]. It also includes use of expensive and frequently unsafe formulation such as injections when less expensive oral formulations would be more appropriate. When antibiotics are indicated, the prescriber should choose the appropriate drug, dose, and duration of effective agent, preferably with the narrowest spectrum and few side effects. Access to affordable health care is limited in many low and middle income countries; hence people rely, to a large extent, on self-medication and buying antibiotics directly from pharmacies, street vendors or markets [4].

Use of treatment guidelines based on clinical presentation is common in developing countries due to unavailability of laboratory services and patient overload. In Tanzania guidelines used are National Standard Treatment Guidelines and the National Essential Medicines List, prepared by Ministry of Health and Social Welfare (URT), World Health Organization pocket book of hospital care for children guidelines for the management of common illnesses with limited resources (WHO), and the Integrated Management of Childhood Illnesses (IMCI), [5–7]. These guidelines have similar recommendations for management of cough and diarrhoea. In the management of diarrhea, all three guidelines recommend use of Oral Rehydration Solution (ORS) and zinc supplements for acute watery diarrhoea (AWD); antibiotics are recommended for management of chronic diarrhoea and dysentery (Includes cotrimoxazole, metronidazole, ceftriaxone and ciprofloxacin). Guidelines classify pneumonia into four categories, namely: no pneumonia (cough/cold), pneumonia, severe pneumonia and very severe pneumonia. This classification is based on the clinical presentation only. Antibiotics are indicated in the management of pneumonia (cotrimoxazole, amoxicillin) and severe pneumonia (ampicilllin and gentamycin). This study assessed the prescribing practice of antibiotics in Mawenzi regional hospital and St. Joseph hospital; we gathered information on how antibiotics were prescribed in treatment of children who presented with cough and/or diarrhoeal, which are among the common childhood presenting complains.

## Methods

This was a cross-sectional, descriptive study. The study was conducted in paediatric wards and outpatients clinics at Mawenzi regional hospital and St. Joseph hospital, in Moshi municipality, Kilimanjaro region – Tanzania. These hospitals receive financial support from the government, hence treatment of under-five is free, and they receive a lot of children from Moshi town and referral from dispensaries around Moshi municipal. According to Mawenzi annual report they attend up to 1500 children and adults with diarrhea and1120 with pneumonia per month in their outpatient clinic. At St. Joseph total attendance per month for under- fives (outpatient) may reach up to 2200 patients. In order to increase the sample size it was important to involve two hospitals.

Sample size was calculated using Epi Info computer software using the Sta Calc calculator for cross-sectional studies with the expected proportion of inappropriate antibiotic prescription of 50% or 0.5 [8]. Probability sampling method was used to recruit children aged 1-59 months whereby all children attending outpatient clinics and the ones admitted with diarrhea and/or cough during the day of data collection at the time of study and meeting the inclusion criteria were consecutively enrolled in the study until the required sample size was attained. Data was corrected once per week in each hospital, on Mondays at Mawenzi regional hospital and on Wednesdays at St.Joseph hospital hence only patients attending on these days were included in the study. These days were chosen as the more children attended the two health facilities. Enrolment was done by the investigator. Hospitalizee children were screened and those meeting the inclusion criteria were enrolled. Outpatients were seen in separate room after being attended by the physician.

Following completion of informed consent, data were collected by interviewing caregivers, using a structured data collection form. Information regarding the place of residence, age, sex, duration of the current episode of diarrhea/cough, consistency of stool, fever, vomiting, history of difficulty in breathing, cyanosis, medical/surgical history, co-morbid conditions, and family/social history was obtained from caretaker. Information on antibiotics prescribed, administration route, dose, and co-administered drugs was obtained from the patient files. Questionnaire was filled by the investigator.

Data were coded and entered into the computer and analysed using SPSS version 11.0. Descriptive statistics were calculated and summarized into frequency tables, charts, and cross tabulations. Statistical significance for categorical variables was tested using the Chi square test at 5% level of significance. Differences with p-values less than 0.05 were considered significant. Estimates of risk were expressed using Odds Ratios (OR) at 95% confidence level. Comparison of antibiotic use in inpatient and outpatient, to estimate how many children who needed /did not need antibiotic but received antibiotic was done (inappropriate prescribing). We also evaluated among those who received antibiotics what percentage got the wrong dose per body weight (inappropriate antibiotic dosage).

Permission to conduct the study was obtained from Mawenzi hospital in-charge, St. Joseph hospital in-charge, and from Regional Medical Officer, Kilimanjaro Region. Ethical clearance was obtained from KCMU-College Research and Ethics Review Committee (certificate no 362). Informed and written consent was obtained from a caretaker accompanying the child.

## Results

A total of 384 children between the age of one month and 59 months were enrolled in the study; 214 (55.7%) from Mawenzi regional hospital and 170 (44.3%) from St. Joseph hospital. The median age of participants was 12 months with interquartile range (IQR) 8 – 18 months. The majority of participants (90.6%) were less than 36 months old. Among the participants, 186 (48.4%) were female and 51.6% were male. Forty seven percent of the participants were outpatient and 53% were inpatient. Fever (60.7%), cough (56.5%), vomiting (51.3%) and diarrhea (50.8%) were the most common complaints (Table 1). On clinical assessment 40.2% had fast breathing and 35.2% dehydration (Table 2).

**Table 1:** Inappropriateness of antibiotic prescribing and patient complaints

Presenting symptoms	Total (n=384)	Prescription status		p-value	OR (95% CI)
		Inappropriate	Appropriate		
		No. (%)	No. (%)		
Fever	233 (60.7)	51 (21.9)	182 (78.1)	<0.001	0.1 (0.08-0.22)
Cough	217 (56.5)	52 (24.0)	165 (76.0)	<0.001	0.2 (0.1-0.3)
Nausea/vomiting	197 (51.3)	105 (53.3)	92 (46.7)	<0.001	3.4 (2.2-5.2)
Watery diarrhoea	195 (50.8)	106 (54.4)	89 (45.6)	<0.001	3.7 (2.4-5.7)
Bloody diarrhoea	9 (2.3)	2 (22.2)	7 (77.8)	0.492	0.4 (0.08-2.09)
Difficulty in breathing	58 (15.1)	4 (6.9)	54 (93.1)	<0.001	0.1 (0.03-0.25)
Abdominal pain	3 (0.8)	2 (66.7)	1 (33.3)	0.565	3.1 (0.3-34.3)
Other	32 (8.3)	11 (34.4)	21 (65.6)	0.529	0.8 (0.4-1.7)

*Patient could have more than one clinical complain, Test used X^2^

**Table 2:** Clinical findings on assessment

Presenting symptom	Total (n=384)	Mawenzi (n=214)	St. Joseph (n=170)
	No. (%)	No. (%)	No. (%)
Respiratory distress	26 (6.8)	15 (7.0)	11 (6.5)
Inability to drink/suck	10 (2.6)	7 (3.3)	3 (1.8)
Lethargy	53(13.8)	37(17.3)	16(9.4)
Stridor when calm	4(1.0)	1(0.5)	3(1.8)
Fast breathing	154(40.2)	84(39.3)	70(41.4)
Definite crackles on auscultation	125(32.6)	71(33.2)	54(31.8)
Dehydration	135 (35.2)	99 (46.3)	36 (21.2)
Some	91 (67.4)	64 (64.6)	27 (75.0)
Severe	44 (32.6)	35 (35.4)	9 (25.0)

*Patient could have more than one clinical finding

There were 199 (51.3%) children who presented with diarrhoea and 230 (59.9%) with cough (45 children had both cough and diarrhea). Of 199 children with diarrhoea, 191 (96.0%) were diagnosed to have AWD, 3 (1.5%) chronic diarrhea and 5 (2.5%) dysentery. Of 230 children presenting with cough, pneumonia was diagnosed in 127 (55.2%) children, severe pneumonia in 42 (18.3%), and cough/cold in 61 (26.5%).

Antibiotics were prescribed to 326 (84.9%) of all children who attended the two study hospitals. Regarding inappropriate prescription based on symptoms, antibiotics prescription was significantly more likely inappropriate for nausea/vomiting and watery diarrhoea (p<0.001; OR=3.4 and 3.7 respectively) and significantly less likely to be inappropriate in fever, cough, watery diarrhoea and difficulty in breathing (p<0.001; OR=0.1, 0.2 and 0.1 respectively Table 1). Also, with reference to concurrent presence diarrhoea and cough, inappropriate antibiotic prescription was significantly more likely to occur in children presenting with diarrhea only (p<0.001; OR=0.1).

Antibiotics were prescribed to 80.9% (n=154) of patients who had AWD and 68.9% (n=61) of those who had cough /cold, while all these children were not supposed to receive antibiotics. Sixty three children (49.6%) with pneumonia and 21 (50.0%) children with severe pneumonia were prescribed antibiotics which are not recommended by the WHO/URT/IMCI to be used as first choice for management of pneumonia.

**Figure 1:**
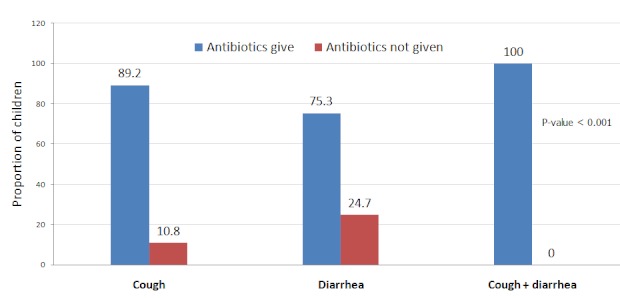
Proportion of antibiotic prescribed according to presentation

Analysis of antibiotics prescription according to clinical presentation showed that 89.2% of 185 children presenting with cough only, 75.3% of 154 children presenting with diarrhea only and 100% of 45 children presenting with both cough and diarrhea antibiotics were prescribed antibiotics. There was higher chance of receiving antibiotics if a child had both cough and diarrhea, the difference in prescription with relation to clinical presentation was statistically significant (p<0.001; Figure 1).

**Figure 2:**
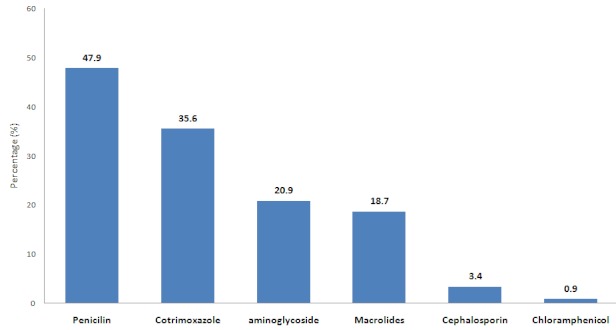
Types of antibiotic prescribed

Antibiotics were prescribed to all children with persistent diarrhoea and dysentery, commonly antibiotic prescribed for diarrhoea were metronidazole, erythromycin and cotrimoxazole. Ninety nine percent of children with pneumonia and severe pneumonia received antibiotics (cotrimoxazole, amoxicillin, gentamycin and ampicillin). Penicillins were the most commonly prescribed antibiotics for children with cough and fever, other drugs were sulphonamides, aminoglycosides, macrolides, chloramphenicol and cephalosporins (Figure 2). Penicillins drugs included amoxicillin, ampicillin, benzylpenicillin, ampiclox, cloxacillin, phenoxmethylpenicillin and flucloxacillin. The only aminoglycides prescribed was gentamycin. Macrolides included azithromycin and erythromycin. Cephalosporins were of first (cephalexin) and third (cefixime, ceftriaxone) generation.

Majorities (71.5%) of children were prescribed only one type of antibiotic; however 21.8% received two types and 6.7% three types. Among 326 children who received antibiotics 179 were inpatient, among those 95 (53%) received one type of antibiotics, 63(35%) two, and 21 (12%) three. Majority of outpatient received one type of antibiotics (94%; 138/147). Inpatients received intravenous antibiotics (94.3%) more often than outpatients (5.7%), and oral antibiotic use was 52.3% for outpatients and 43.7% for inpatients.

Inappropriate prescription was more frequent among clinical officers than assistant medical officers and medical officers (48.9% vs. 30.1% and 13.0% respectively; Table 3). With reference to medical officers, clinical officers and assistant medical officer were significantly more likely to prescribe antibiotics inappropriately (p<0.05; OR = 0.2, 0.4 respectively Table 3). Inappropriate antibiotic dosage (giving too high or too low dose according to the weight of a child) for treatment of pneumonia was common at both hospitals and among prescribing clinicians (at least 79% (255/322) children were given inappropriate dosage). With reference to medical officers, clinical officers were significantly more likely to prescribe wrong dosage (p<0.001, OR= 0.2 Table 3). Apart from antibiotics, children with diarrhoea also received ORS (68.4%), zinc supplementation (28.0%), intravenous fluids (42.2%) and mebendazole (8.5%) and 30.4% of children with cough were also given cough syrup.

**Table 3:** Appropriateness of antibiotic prescribing and dosage in under five according qualification of prescriber

Variable	Total	Inappropriate prescription	Appropriate prescription	p-value	OR (95% CI)
**Qualification of prescriber:**					
Clinical Officer	235	115 (40.4)	170 (59.6)	0.001	0.2 (0.2-0.6)
Assistant Medical Officer	103	31 (30.1)	72 (69.1)	0.026	0.4 (0.1-1.0)
Medical officer	46	6 (13.0)	40 (87.0)	Reference	
Presenting complaint:					
Cough	185	43 (23.2)	142 (76.8)	0.073	0.4 (0.2-1.1)
Diarrhea	154	104 (67.5)	50 (32.5)	<0.001	0.1(0.0-0.2)
Cough + Diarrhea	45	5 (11.1)	40 (88.9)	Reference	
		**Inappropriate dosage**	**Appropriate dosage**		
**Qualification of prescriber:**					
Clinical Officer	204	177 (87.2)	26 (12.8)	<0.001	0.2 (0.1-0.5)
Assistant Medical Officer	92	60 (67.4)	29 (32.6)	0.542	0.7 (0.3-1.7)
Medical officer	30	18 (60.0)	12 (40.0)	Reference	

## Discussion

The present work at Mawenzi and St. Joseph hospitals aimed at assessing antibiotic prescribing practice in children aged one to 59 months. Antibiotics were prescribed to almost 85% of under-five who had cough and/or diarrhoea attending the two hospitals during the period of the study. This proportion is similar to that reported in other studies where the proportion use of antibiotics ranged from 72.2% to 81.1% in Nepal (children less than 13years), India (adults) and Vietnam (under fives) [9–11]. Excessive use of antibiotics has also been reported in developed countries but less than in developing countries e.g. 51% of adults received unnecessary antibiotics for cough in the United States of America [12].

The use of antibiotics was high in all illness categories including conditions for which antibiotics were not recommended by WHO [6], like common cold and diarrhoea. This finding was similar to studies from Nepal and Vietnam [9, 11]. Misuse of antibiotics is due to diagnostic uncertainty, lack of prescriber knowledge, lack of opportunity for follow-up, easy availability of antibiotics and lack of treatment guidelines [1].

The majority of the patients (71.5%) who received antibiotics got only one kind of antibiotic. This is consistent with a survey in four regions of Tanzania (Mwanza, Mbeya, Kilimanjaro and Dar es Salaam), where in Kilimanjaro, the majority received only one type of antibiotic and less than 6% received more than one antibiotic [14]. Some of in-patients received two (35.2%) or three (11.7%) antibiotics, however this is not necessarily bad practice as in some patients you need to give more than one type of antibiotic.

In this study, penicillins, sulphonamides, aminoglycosides and macrolides were commonly prescribed from the two hospitals, chloramphenicol and cephalosporins were less common prescribed. Penicillins and cotrimoxazole have also been reported to be commonly used antibiotics in other developing countries like Nigeria, Croatia and Peru [15–17]. In our study, 68.9% of children who had common cold were prescribed antibiotics. According to WHO and URT treatment guidelines children with common cold are not supposed to receive antibiotics. The Baseline Survey of the Pharmaceutical Sector in Tanzania of 2002 to assess the availability and use of medicines in Tanzania revealed that antibiotics were used by more than 90% of patients in management of non-pneumonic acute respiratory infections [14]. In Peru, 58% of children who had cough /cold and diarrhoea were prescribed antibiotics by nurses and doctors [17]. Antibiotics do not reduce the severity or time of illness in viral infections hence their use exposes patients to risks of medicine use without benefit. Ninety nine percent of children with pneumonia and severe pneumonia in this study appropriately received antibiotics. When children with pneumonia are treated promptly and effectively with antibiotics their chance of survival increases significantly. Common antibiotics prescribed for pneumonia were penicillins, sulphonamides, and aminoglycosides. Amoxycillin and sulphonamides were the most prescribed drugs in management of pneumonia in other areas too e.g. rural Peru and Kibaha-Tanzania [17, 18]. Children with severe pneumonia were most of the time given intravenous ampicillin or benzyl penicillin for the first 48 hours and then changed to amoxicillin or cotrimoxazole. Ampicillin, amoxycillin, and cotrimoxazole are recommended by treatment guidelines. Although it was appropriate to prescribe antibiotics, some received antibiotics that are not recommended by treatment guidelines e.g. erythromycin, azithromycin, and cephalosporin. These drugs are more expensive and they are not indicated in the WHO, 2007, IMCI, 2004, URT, 2009 guidelines as the first line drugs of management of pneumonia. This is consistent with studies from Vietnam and Nigeria where cephalexin was more commonly prescribed than the drugs recommended by guidelines [11, 15].

In our study, antibiotics were prescribed to 80.6% children with AWD, antibiotics have no role in the management of AWD as most of the time its cause is viral. Our findings are consistent with reports from other low income countries where 68-95% patients with diarrhea received antibiotics [11, 13]. Patients who had persistent diarhoea and dysentery were properly given antibiotics, as recommended by the treatment guidelines.

Prescribing of antibiotics to children who did not need antibiotics was done by all prescribers, though it was higher with clinical officers than with assistant medical officers and medical officers. This shows that there is less irrational prescribing with higher qualifications. This was also observed in India where there was less antibiotic use in specialist practices with higher qualifications staffs and staff with better opportunities for updating knowledge [10]. In the rural Peru survey, there was no difference in the prescription of antibiotics for cough and diarrhoea among nurses and doctors, but less prescribing of antibiotics by trained pharmacists [17]. This shows that rationale prescribing would improve if there is continuing medical education to all prescribers.

Most of the children (79%) received inappropriate dosage (too high or too low). The proportion of inappropriate antibiotic dosage was statistically different among prescribers with different qualifications. This was different with the findings from Netherlands were 97.6% of children received correct dose; this might be due to the different in qualification of the clinicians [18].

Our study had some limitation, the assessment of inappropriate prescribing might be overestimated because some of the patients who had secondary bacterial infection, might have been treated appropriate. Also clinician might have behaved differently when they know that they are being observed, hence some of the clinicians might have prescribed more carefully.

## Conclusion

This study observed a high antibiotic prescription rate by clinicians and that the treatment guidelines for management of patients who presented with cough and/or diarrhoea were not followed. Updating health training programmes for clinicians on how to prescribe medicines e.g. continuing education on disease management would help in reducing irrational prescribing practices.
